# Supplementary material for: Isolation and Identification of Endophytic Bacterium B5 from Mentha haplocalyx Briq. and Its Biocontrol Mechanisms Against Alternaria alternata-Induced Tobacco Brown Spot
Source: J Fungi (Basel). 2025 Jun 12;11(6):446. doi: 10.3390/jof11060446 (PMC12194751; doi:10.3390/jof11060446)
Supplement: Supplementary file 1 [file jof-11-00446-s001.zip › jof-3646039-supplementary.pdf]

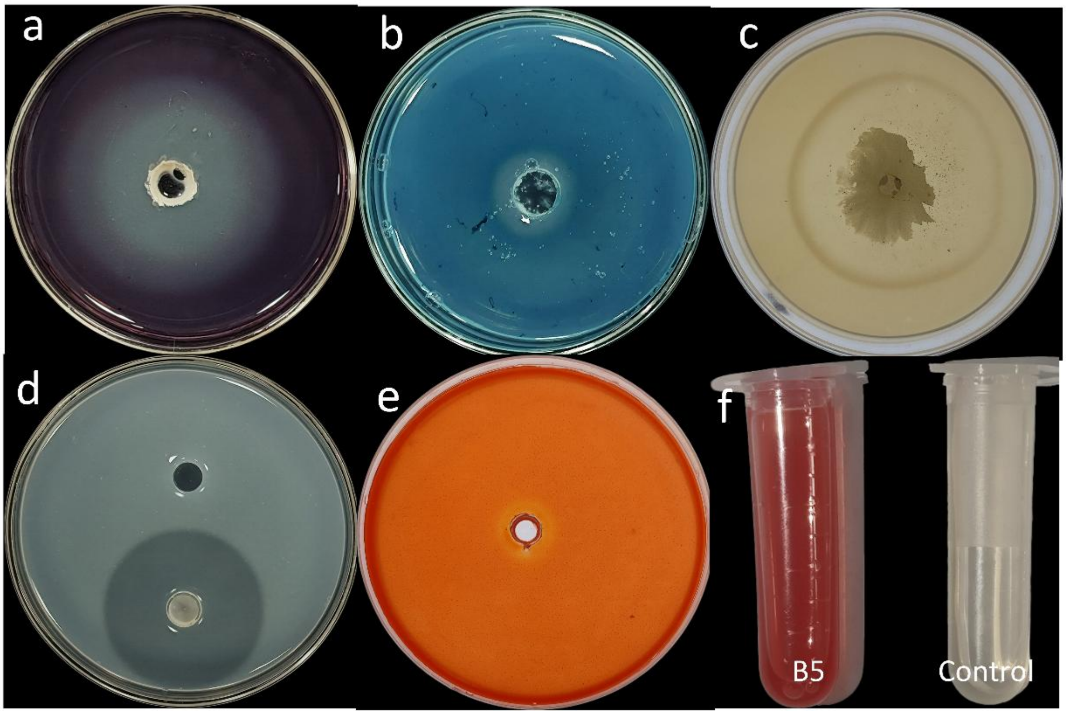


**Figure S1.** Detection of cell wall-degrading enzymes, siderophores, and indole-3-acetic acid (IAA):(a) Amylase activity, (b) Siderophore production, (c) Glucanase activity, (d) Protease activity, (e) Cellulase activity, (f) IAA production.

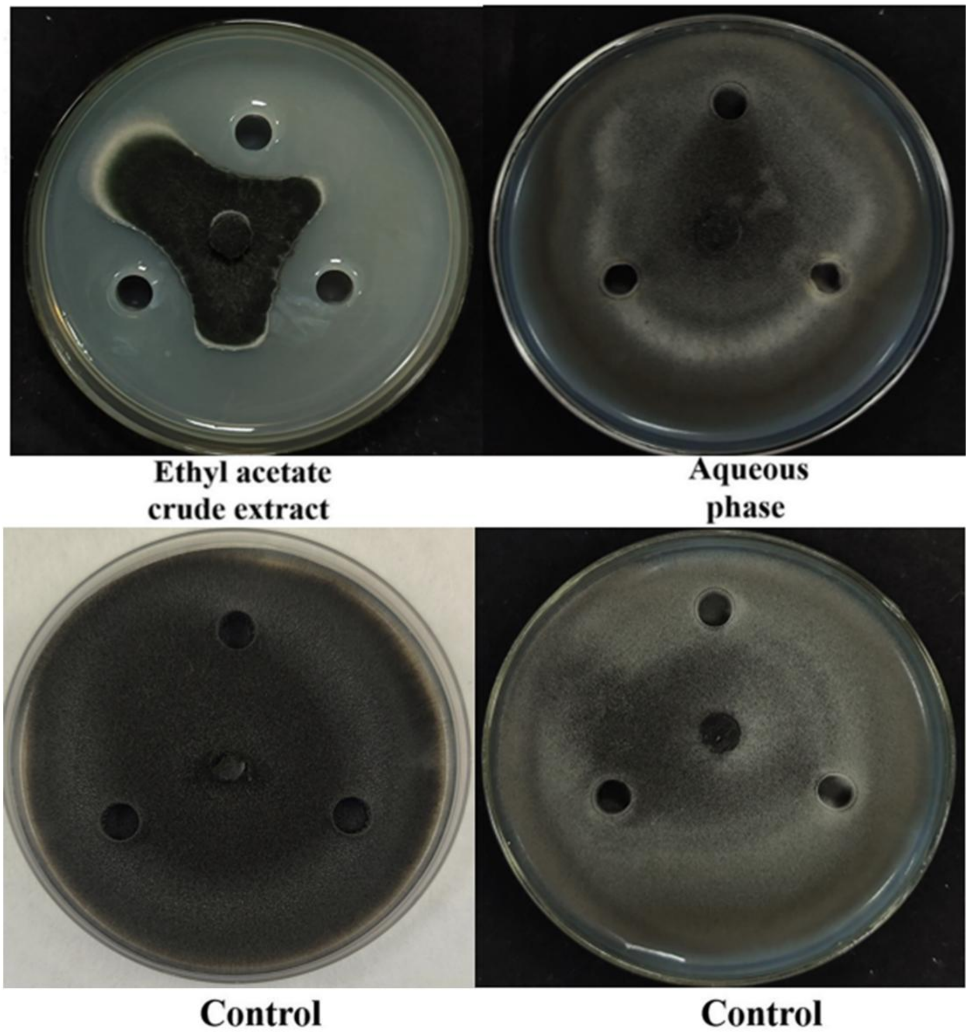


**Figure S2.** Inhibitory activity of strain B5 crude extracts against the growth of *A. alternata*. The ethyl acetate fraction and aqueous fraction of the crude extracts (10%, v/v) were added to PDA medium and incubated at 25°C for 7 days. Pathogen-inoculated PDA medium (without extracts) served as the control.

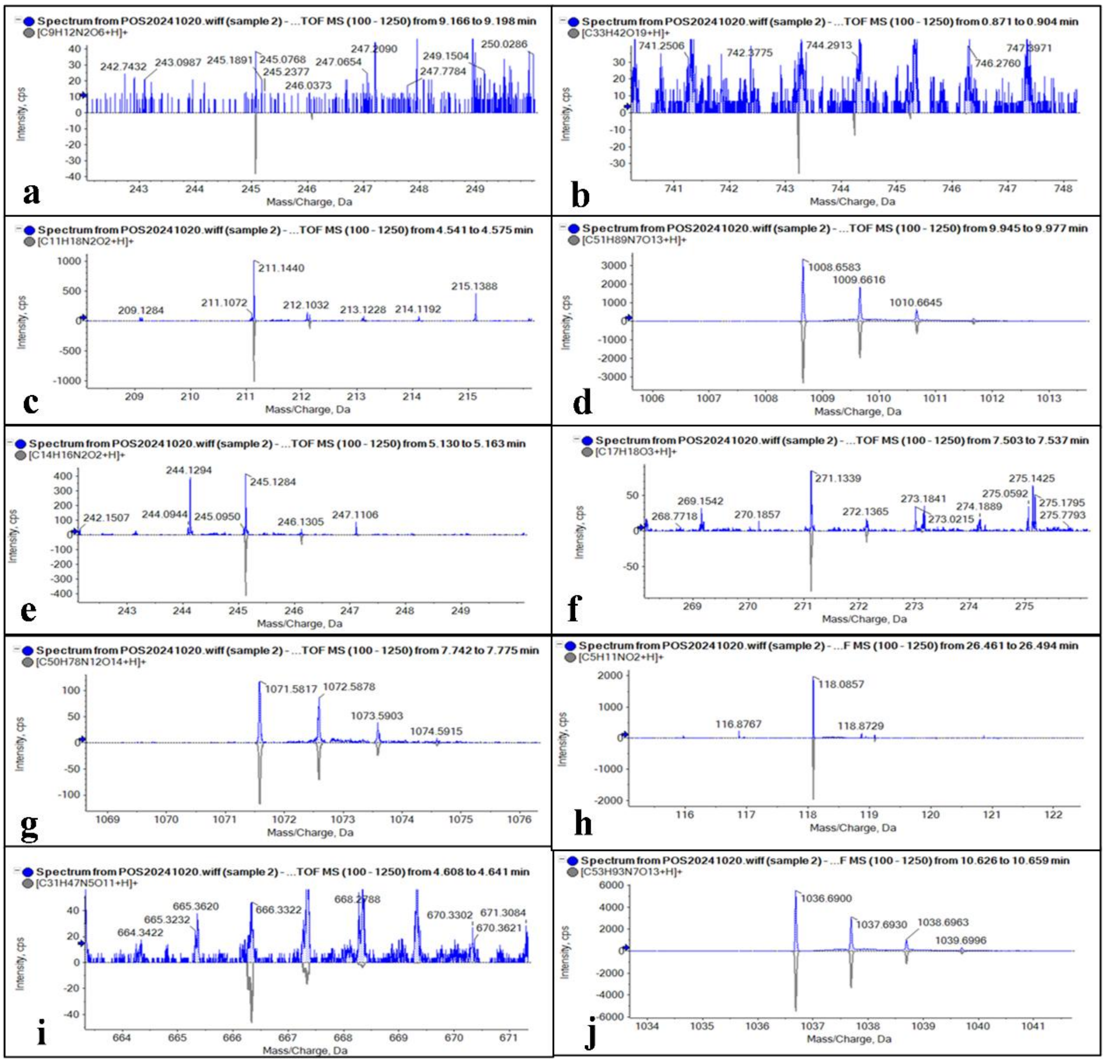

**Figure S3**. Mass spectrometry analysis of lipopeptide bioactive compounds produced by strain B5.Identified compounds: (a) Uridine; (b) Toxenutin; (c) Cyclo(Proline-Leucine) dipeptide; (d) Surfactin A; (e) Cyclo(D-Phenylalanine-L-Proline); (f) 4',5-Trimethoxy-trans-stilbene; (g) Iturin A-7; (h) Betaine; (i) Bacillaene; (j) Surfactin C.
